# Differences in Training Adaptations of Endurance Performance during Combined Strength and Endurance Training in a 6-Month Crisis Management Operation

**DOI:** 10.3390/ijerph17051688

**Published:** 2020-03-05

**Authors:** Kai Pihlainen, Keijo Häkkinen, Matti Santtila, Jani Raitanen, Heikki Kyröläinen

**Affiliations:** 1 Training Division, Defence Command, P.O. Box 919, 00131 Helsinki, Finland; 2 Neuromuscular Research Center, Faculty of Sport and Health Sciences, University of Jyväskylä, P.O. Box 35 (VIV), 40014 Jyväskylä, Finland; keijo.hakkinen@jyu.fi (K.H.); heikki.kyrolainen@jyu.fi (H.K.); 3 Department of Military Pedagogy and Leadership, National Defence University, P.O. Box 7, 00861 Helsinki, Finland; matti.santtila@kolumbus.fi; 4 Faculty of Social Sciences (Health Sciences), Tampere University, P.O. Box 100, 33014 Tampere, Finland; jani.raitanen@ukkinstituutti.fi; 5 UKK Institute for Health Promotion Research, P.O. Box 30, 33501 Tampere, Finland

**Keywords:** soldier, combined training, cardiorespiratory fitness, bioimpedance, training response, adaptation

## Abstract

Decreases in aerobic fitness during military operations have been observed in several studies. Thus, differences in training adaptations during a 6-month crisis-management operation were compared by using the change in endurance performance as the outcome measure. Sixty-six male soldiers volunteered for the study, consisting of pre–post assessments of blood biomarkers, body composition, physical performance, and the military simulation test (MST) performance. Physical training volume was self-reported. After the follow-up, the data were divided based on individual changes in endurance performance. Endurance performance was improved in the high-responder group (HiR, n = 25) and maintained or decreased in the low-responder group (LoR n = 24). During the operation, the LoR group decreased while the HiR group increased their endurance training frequency from the pre-deployment level (Δ 28 ± 57% vs. −40 ± 62%, *p* = 0.004). Fat mass decreased (−7.6 ± 11.7% vs. 14.2 ± 20.4%, *p* < 0.001), and 1-min push-up (27.7 ± 21.9% vs. 11.7 ± 26.1%, *p* = 0.004) and MST performance improved (−13.6 ± 6.8% vs. −7.5 ± 6.5%, *p* = 0.006) more in the HiR group. No differences were observed in the changes of other physical performance test results or analyzed biomarkers. In conclusion, soldiers who were initially leaner and fitter in terms of lower body strength and power were more likely to decrease their aerobic fitness during the operation.

## 1. Introduction

The demands of operative duties constitute the basis for the development and maintenance of the physical performance of soldiers [1,2]. Typical military tasks such as marching, digging, manual material handling [1,2] are often performed in a prolonged manner, combined with environmental stress factors, which might accumulate fatigue in soldiers. Furthermore, soldiers commonly perform their operative duties wearing combat gear and carrying other equipment which might have negative impacts on job performance in relation to the weight of the carried load [3,4]. Thus, optimal occupational performance of a soldier requires a high level of combined strength and aerobic fitness.

Based on the requirements of military work, the development and maintenance of physical performance of soldiers should include combined strength and endurance training [5,6]. Aerobic fitness is an important contributor to optimal performance, in numerous military simulations of varying durations, both from the performance and recovery perspective [7]. Habitual endurance training has been shown to improve aerobic fitness through central (e.g., increased stroke volume) and peripheral (e.g., increased mitochondrial content) adaptations [8,9,10,11]. In addition, evidence from the literature suggests that improvements in neural [12,13] and hypertrophic pathways [14,15] lead to increases in muscle strength which might be a crucially important component of soldiers’ physical performance, especially during intensive combat situations [16]. In certain tense situations, soldiers are required to rush and sprint short distances, interspersed with recovery periods [17,18]. The speed of such sprints has been associated with muscle strength and the power of the lower extremities [16]. All of the above-mentioned variables of occupational performance are modifiable through regular physical training. In a military environment, combined strength and endurance training might be a time-efficient method to simultaneously improve aerobic and muscle fitness [6,19]. Despite the known benefits of physical performance enhancement, studies focusing on combined strength and endurance training adaptations during a military operation are limited.

Physical stress induced by military field exercises has been documented extensively. For example, Ojanen et al. [20] observed deteriorated physical performance and hormonal balance in soldiers, during and after a three-week military field exercise. The results are well in line with an earlier study showing that an 8-week Army Ranger Course induced negative energy balance and >10 kg average weight loss, accompanied with decreases in serum testosterone, insulin-like growth factor-1 (IGF-1), and increases in cortisol (COR) concentrations [21]. In addition to military training, only a few studies have shown that international military operations might deteriorate physical performance, especially aerobic fitness, and could induce undesirable changes in body composition, such as an increase in fat mass [22]. These changes compromise occupational performance [7,23], increase a risk of injuries [24] and thereby, have negative impact on the mission readiness of soldiers. 

Taken together, the physical performance of soldiers should be at a high level before military operations, as the physiological homeostasis, and thereby, the optimal status for the maintenance of fitness might be disturbed under tense operative circumstances. Nevertheless, especially during longer deployments, soldiers should engage with regular physical training in order to maintain their readiness for unexpected changes in security situations. Therefore, the purpose of the present study was to investigate differences in training responses and adaptations of endurance performance during combined strength and endurance training in a six-month crisis management operation in the Middle East.

## 2. Materials and Methods 

**Figure 1:**
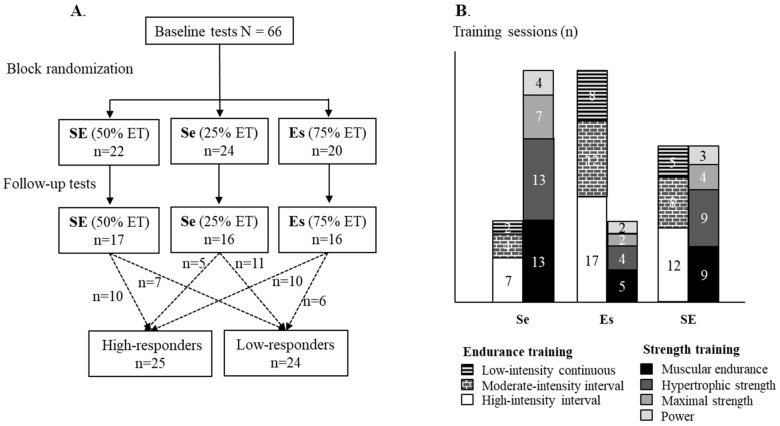
Study design (**A**) and the strength and endurance training plan of the groups (**B**). Se = strength emphasized training group; Es = endurance emphasized training group; SE = evenly balanced strength and endurance training group; and ET = endurance training.

Endurance performance adaptations to combined strength and endurance training were studied during a crisis-management operation in Southern Lebanon. Baseline body composition, physical performance, and serum biomarkers were studied before block-randomizing [25] the soldiers into three training groups (Figure 1A). The training groups were provided a standardized combined strength and endurance training program to be performed twice a week. Depending on the program, strength and endurance training frequency was set to either 1 + 3 (75% endurance training), 2 + 2 (50% endurance training), or 3 + 1 (25% endurance training) sessions in two weeks (Figure 1B). In addition, the soldiers were encouraged to maintain their habitual training frequency at the level of pre-deployment and to adjust their emphasis on the strength and endurance training to the given program. The training was self-reported by using training diaries. In addition, the soldiers were interviewed before and during the operation for achieving a better view of their training. The follow-up tests were performed five months after the baseline measurements. During the study, the soldiers performed their operative duties including typical military tasks, such as patrolling and observing outside the military base, as well as maintenance and headquarter duties inside the base. Recently, a more detailed description of the physical activity and work load [26] of the participants as well as their diet [27] has been published. 

Sixty-six voluntary male soldiers who were deployed for a crisis management operation in the Middle East took part in the baseline measurements. Before the deployment, the soldiers were examined by a physician. The exclusion criteria for deployment included health limitations with a need of permanent medication and aerobic fitness level lower than 2300 m in the 12-min running test [28]. The study was approved by and conducted in accordance with the statement of the Ethics Board of the Central Finland Health Care District (KSSHP E1/2013). The soldiers were informed of the benefits and risks of the investigation prior to signing an institutionally approved informed consent document to voluntarily participate in the study. 

The baseline means ± standard deviations (SD) with the range for age, height, weight, body mass (BM), and body mass index (BMI) of the participants were 29.8 ± 8.5 (20.4-51.2) years, 180 ± 7 (165-199) cm, 79.4 ± 8.2 (58.5-105.6) kg, and 24.5 ± 2.3 (21.1-32.8) kg/m^2^, respectively.

The baseline measurements were carried out after two weeks of non-standardized acclimatization inside a military base in South-Lebanon. The measurements were repeated accordingly after the 5-month follow-up. The soldiers wore light underwear in the body composition measurements and shorts, and T-shirt and running shoes in the tests of endurance and neuromuscular performance. During the first day of the measurements, body composition measures and blood sampling were conducted in the morning, followed by the measurements of maximal strength in the evening. Thereafter, the soldiers were provided a minimum of 15 min for recovery before the muscle endurance tests. The assessment of strength, endurance, and military specific performance were performed on separate days, with a minimum of 24 h between the tests. 

Assessment of body composition and blood sampling were performed in a military hospital in the morning after a 10-h overnight fast. Body height was measured by using a wall-mounted height board (Seca Bodymeter 206, Seca GmbH & Co, Hamburg, Germany). BM, skeletal muscle mass (SMM), and fat mass (FATM) were determined by using the segmental multi-frequency bioimpedance analysis (InBody 720, Biospace, Seoul, South Korea), in accordance with the guidelines of the manufacturer.

Blood samples were drawn from the antecubital vein and serum was separated from the blood using a centrifuge (1000 rpm, 8 min). The samples were frozen below −20 °C for further transportation and analysis. Assays for serum TES, sex-hormone binding globulin (SHBG), COR, and IGF-1 were performed by Immulite 2000 XPi (Siemens Healthcare, Llanberies, UK), using commercial chemiluminescent enzyme immunoassay kits, according to the manufacturer’s guidelines. The inter-assay coefficients of variance (CV) for assays of TES, SHBG, COR, and IGF1 were 7.0%–7.2%, 4.5%–6.2%, 4.6%–5.8%, and 3.7%–7.4%; and that of sensitivity was 0.5, 0.02, 5.5 nmol·L^−1^, and 2.6 pmol·L^−1^, respectively.

Maximal isometric force of the lower and upper extensor muscles was measured bilaterally in a sitting position, using the electromechanical dynamometer [29] (University of Jyväskylä, Jyväskylä, Finland). In the lower extremity test, the seat was set to maintain knee and hip angles of 107° and 110°, respectively. In the upper extremity test, the handle bar was adjusted to the height of shoulders and the seat was set to maintain an elbow angle of 90°. The soldiers were instructed to exert their maximal force in all three trials, which were separated by a minimum of 30 s for recovery. The best performances with regard to maximal force output were selected for further analysis.

Maximal standing long jump (*SLJ*) was used to assess the maximal power production of the lower extremities [30]. The soldiers were familiar with the test since the same method has been used during their basic military training period. Before the three test attempts, the soldiers were provided with instructions on how to perform the jumps with the optimal technique preceding five to seven warm-up trials. The jumps were performed from a standing position, feet at pelvis to shoulder width apart on rubber mattresses designed for the purpose (Fysioline Co., Tampere, Finland). Explosive bilateral take-off was assisted by a powerful swinging of the arms and extension of the hip. The landing was performed bilaterally, and falling backwards led to a disqualification of the attempt. The result of the best jump was expressed as centimeters of the shortest distance from the landing point to the starting line.

Sit-up, push-up, and pull-up tests were used to assess the dynamic muscle endurance capacity of the trunk and upper extremities. A test supervisor showed the correct performance technique before each test. The soldiers were also informed that after a notice from the supervisor, incorrect repetitions would not be calculated to the test result.

Sit-ups were used to assess performance of the abdominal and hip flexor muscles [31]. In the starting position of the sit-up test, the soldier laid on his back, while his knees were bent at a 90° angle, elbows pointing upwards, and fingers interlocked behind the head. The ankles were supported by an assistant to keep the heels in contact with the ground during the test. From the starting position, the upper body was raised forward with the trunk muscles until the elbows reached the knee-level. One repetition was completed when the body was lowered until the bottom of the shoulder blades touched the ground. The test result was expressed as a number of consecutive repetitions in 60 s. 

The push-up test was to evaluate performance of the arm and the shoulder extensor muscles [32]. The correct position for the push-up test was determined while the soldier was lying on the floor in a front-leaning rest position, feet parallel at pelvis-to-shoulder width and hands positioned so that the thumbs could reach the shoulders while the other fingers pointed forward. From this position, the soldiers were instructed to take the starting position by extending their arms straight, while keeping the body in a straight line from the shoulders to the ankles and maintaining the knee and hip angles steady, throughout the test. One repetition was counted when the soldier lowered his torso by bending his elbows until the upper arms were parallel to the floor and returned to the starting position by extending his arms. The test result was expressed as the number of consecutive correct repetitions during 60 s.

The pull-up test was used in order to measure the performance of the arm and shoulder flexor muscles. In the starting position of the pull-up test, the soldiers were hanging from a horizontal bar with an underhand grip, keeping the arms and feet straight. One repetition was performed when the body was raised by flexing the arms from the starting position until the chin exceeded the height of the bar level. The hip and legs were instructed to be extended throughout the test. The result of the test was expressed as the number of consecutive repetitions, until volitional exhaustion.

Aerobic endurance performance was assessed using the 3000-m running test (3000-m). Due to the time and logistical constraints, it was not possible to perform the direct assessment of aerobic capacity (e.g., oxygen consumption measurements) in the military base. The 3000-m test was performed on a standardized 1-km track covered with asphalt. The total ascent and descent of the track was 32 m. The soldiers were instructed to complete the test with maximal effort and in the shortest possible time. The duration of the test was recorded with a stopwatch (Select Sport, Glostrup, Denmark), while the heart rate was recorded by using chest-strapped monitors (Memory belt, Suunto, Vantaa, Finland) and analyzed with computer analysis software (Firstbeat PRO, Firstbeat Technologies, Jyväskylä, Finland).

Occupational physical performance and the anaerobic capacity of the soldiers was assessed by the military simulation test (MST) [23], which was designed to assess military-specific, high-intensity performance of crisis-management soldiers. The MST consisted of typical army soldier maneuvers (rushes, jumps, changes in movement directions, crawling) and tasks (load carriage, casualty drag) which might be performed in an ambush during a patrol or transport at the deployment area. The total length of the MST track was 243 m. The test was performed in the shortest possible time wearing a combat dress uniform, leather boots, and combat gear, including a body armor, helmet, and replica assault rifle. The total weight of the combat load, including the weapon replica, was 22.5 ± 1.0 kg. The performance time was recorded with a stopwatch (Select Sport, Glostrup, Denmark).

To assess the differences in habitual strength and endurance training before vs. during the operation, the soldiers were interviewed six weeks before the deployment, inquiring their endurance and strength training frequency from the preceding two months. The soldiers were asked “on average, how many times per week have you performed endurance-type of training, e.g., walking, running, swimming, cycling, during the preceding two months?” Similarly, for strength training, the soldiers were asked “on average, how many times per week have you performed strength-type of training, e.g., gym training, weight lifting, during the preceding two months?” The interview was repeated at the deployment area during the post measurements.

After the baseline measurements, the soldiers were randomly allocated to one of the three combined strength and endurance training groups. Training was recorded using the self-reported training diaries. The diaries of the three intervention groups included a progressive combined strength and endurance training program with illustrated instructions of the exercises. The actual exercises of all intervention groups were similar but the strength-to-endurance training ratio in the three groups varied between the groups, as mentioned earlier. For example, the training diary of the SE group consisted of two strength and two endurance training sessions in two weeks, while the diary of the Se group consisted of three strength training sessions and one endurance training session. Altogether, the training program included 50 standardized strength and endurance training sessions (Figure 1B). All exercises were demonstrated and practiced before the initiation of the intervention. Intensity and volume were determined individually for strength training. For hypertrophic and maximal strength training, the soldiers were instructed to select weights for each exercise so that the last predetermined repetitions in each set would proceed as close to concentric failure as possible. For endurance exercises, the peak heart rate was determined from the highest measured heart rate during the 3000-m run, utilizing the Firstbeat PRO analysis (Firstbeat Technologies, Jyväskylä, Finland). The soldiers were provided with a heart rate monitor for endurance training (M1, Suunto, Vantaa, Finland). Due to the nature of the operation, the soldiers performed the exercises without supervision. Despite the twice-a-week programming, the soldiers were encouraged to maintain the weekly training frequency, which they were accustomed to preceding the operation, but had to adjust the strength-to-endurance training ratio to match the program of their allocated group.

At the end of the follow-up, the training diaries were collected and analyzed. The available training data were analyzed for the relative strength and endurance training frequency (sessions/week). In addition, endurance training was analyzed for volume (minutes/week) of different intensity zones (low < 75% HR_peak_, moderate 75–85 HR_peak_, high-intensity > 85 HR_peak_), and strength training for the lower and upper body volume load (kg/week). The training diary statistics for each group are presented in the supplemental material (Supplement Table S1).

**Figure 2:**
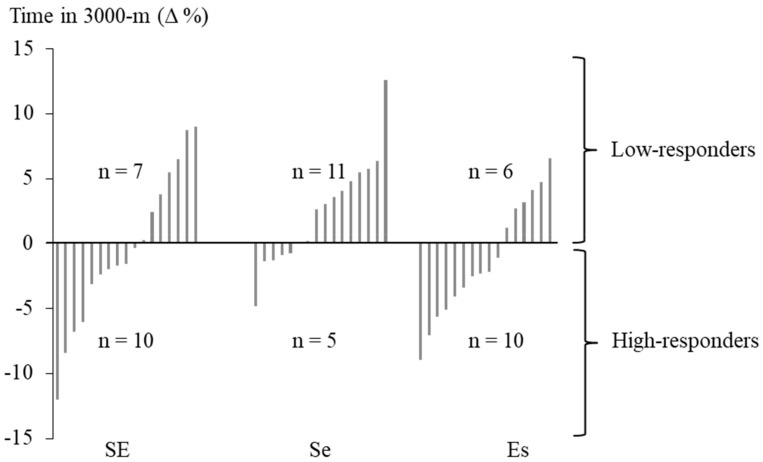
The classification into high-responders and low-responders. Soldiers who decreased their 3000-m running test time were termed high-responders, while the low-responders either maintained or increased their running test time during the operation.

Out of the 66 soldiers who initially took part in the study, the data were analyzed for those who participated in the 3000-m running test at the beginning and at the end of the operation (n = 49). The combined data of these soldiers were re-grouped to high responders (HiR, n = 25) and low responders (LoR, n = 24), according to the changes in endurance performance assessed by the 3000-m running test (Figure 2). The HiR group consisted of soldiers who decreased their 3000-m test time, while the soldiers in the LoR group either maintained or increased their running test time during the operation. Descriptive statistics (mean ± SD) were reported when appropriate. The relative changes were calculated on the basis of individual values. The significances of group differences were tested by using the Mann–Whitney test. In addition, the relationships between relative changes of the measured variables were tested with Spearman’s rank correlation coefficient using all available data. IBM SPSS Statistics version 25 (Chicago, IL, USA) was used for all statistical analyses. The *p* < 0.05 was used to establish statistical significance.

## 3. Results

**Figure 3:**
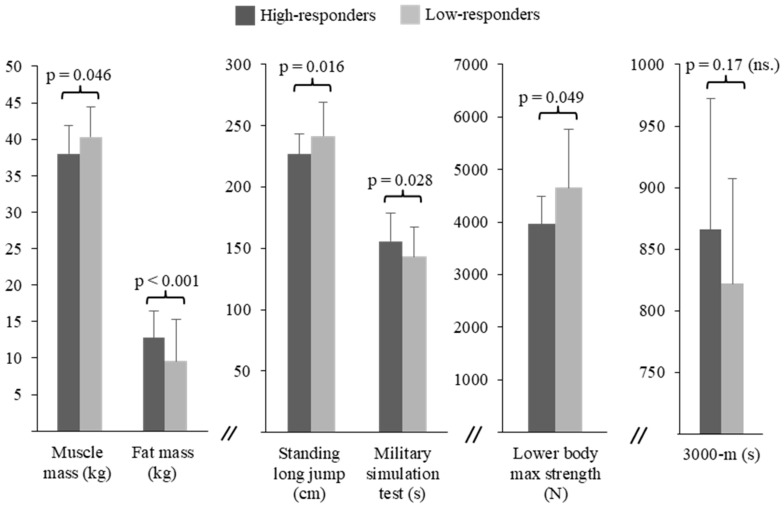
Comparison of body composition and physical performance between the high-responders and low-responders for endurance performance at baseline. ns.—non-significant.

More than half (51%) of the soldiers improved their endurance performance and, thus, they were HiR in terms of combined strength and endurance training adaptation (Figure 2). Before the operation, no differences were observed in the endurance training frequency between the HiR and LoR groups, while the LoR group performed strength training more frequently than HiR (Mean ± SD: 1.8 ± 1.4 vs. 2.9 ± 1.2 times/week, *p* = 0.008). At baseline, the mean 3000-m test times of the HiR and the LoR groups did not differ (866 ± 106 vs. 822 ± 85 s, *p* = 0.17). Significant baseline differences between the HiR and LoR groups (Figure 3) were observed in SMM (38.0 ± 3.9 vs. 40.3 ± 4.1 kg, *p* = 0.046), FATM (12.8 ± 3.6 vs. 9.6 ± 5.7 kg, *p* < 0.001), maximal strength of the lower extremities (3959 ± 532 vs. 4564 ± 1116 N, *p* = 0.049), SLJ (227 ± 16 vs. 242 ± 27 cm, *p* = 0.016), and MST (156 ± 23 vs. 143 ± 24 s, *p* = 0.028). In addition, a trend for the lower baseline 1-min push-up test result of the HiR group (37 ± 12 vs. 44 ± 13 reps/min, *p* = 0.053) was observed. Group comparisons at baseline for all variables are presented in Table 1.

The training diary statistics showed that the HiR group performed their strength training of the lower body with a lower average volume (e.g., total amount of lifted weight/week) than the LoR group (14354 ± 6076 vs. 19489 ± 6202 kg/week, *p* = 0.010). In addition, a trend for a lower average strength training frequency in the HiR group (1.3 ± 0.7 vs. 2.1 ± 2.4 sessions/week, *p* = 0.052) was observed. 

**Figure 4:**
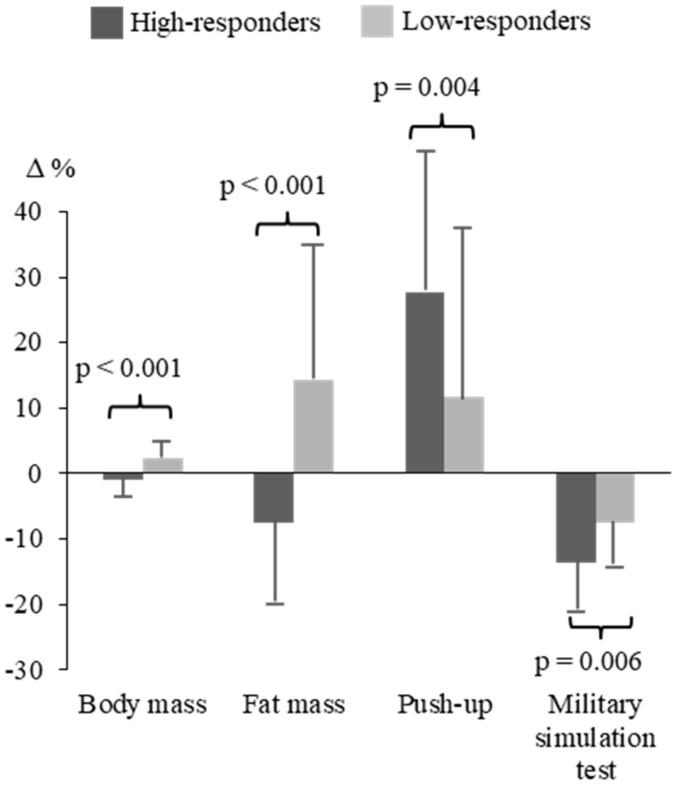
Comparison of differences in relative changes in variables with statistically significant group difference between the high-responders and low-responders of endurance performance.

Significant differences in the relative changes of the measured body composition and physical fitness variables during the operation, favoring the HiR group (Figure 4), included BM (−1.0 ± 2.5% vs. 2.3 ± 2.8%, *p* < 0.001), FATM (−7.6 ± 11.7% vs. 14.2 ± 20.4%, *p* < 0.001), 1-min push-up (27.7 ± 21.9% vs. 11.7 ± 26.1%, *p* = 0.004), and MST (−13.6 ± 6.8% vs. −7.5 ± 6.5%, *p* = 0.006). In addition, interview-based training frequency revealed a relative decrease in endurance training (−40%) in the LoR group, while the HiR group increased their endurance training by 28% (group comparison, *p* < 0.001). The comparison of the training as well as relative changes in all available variables between the HiR and LoR groups is presented in Table 2.

In the total group of participants, the increase in the average strength training frequency correlated with the relative increase in BM (*r* = 0.42, *p* = 0.004), SMM (*r* = 0.31, *p* = 0.036), and FATM (*r* = 0.35, *p* = 0.018). In addition, the increase in the strength-to-endurance training ratio (%) correlated with the relative increase in BM (*r* = 0.43, *p* = 0.034) and also, a trend for decreased endurance performance (strength-to-endurance training ratio vs. 3000-m, *r* = 0.33, *p* = 0.065) was observed. 

**Figure 5:**
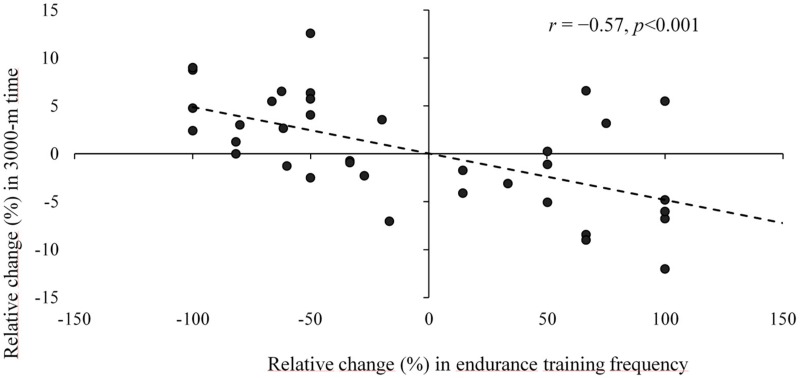
Relative increase in weekly endurance training frequency during the deployment vs. pre-deployment, plotted against relative reduction in 3000-m time (*r* = −0.57, *p* < 0.001).

The relative increase in the weekly endurance training frequency during the deployment vs. pre-deployment correlated (*r* = −0.57, *p* < 0.001) with the relative reduction in 3000-m time (Figure 5). The relative increase in 3000-m time correlated with the respective increase in BM (*r* = 0.41, *p* = 0.004), as well as FATM (*r* = 0.53, *p* < 0.001). Finally, the relative increases in the MST time correlated with the respective increases in the 3000-m time (*r* = 0.48, *p* < 0.001).

## 4. Discussion

The present study showed that despite the similar endurance performance at baseline, soldiers who were more likely in a risk of decreasing their aerobic fitness, e.g., the LoR group, were initially leaner and they had a higher physical performance in terms of lower body strength and power. In addition, the LoR group was not able to maintain the average endurance training frequency at the level preceding the operation. Additionally, increased FATM was observed in the LoR group, whereas the HiR group decreased FATM during the operation. Relative increases in the 3000-m time correlated with respective increases in BM and FATM. Finally, the LoR group was not able to improve 1-min push-up and the MST performance to the same extent as the HiR group. From a physical performance perspective, many of these changes in the LoR group might reflect a reduction in military readiness, which is not desirable during the operation and should be avoided by providing more individualized strength and endurance training programs, during deployment. In addition to the operative task analysis, individualization should consist of factors like baseline physical performance, strength training and endurance training history, and body composition of soldiers.

Aerobic fitness seems to be an important component of soldiers’ physical performance during prolonged physical activities, with extra loads (e.g., marching [7]) and intensive combat situations (e.g., rushes, casualty evacuation [7,23]). Aerobic fitness can be affected by endurance training, which leads to central and peripheral adaptations [8,9,10,11]. Low intensity endurance training increases the mitochondrial density and cellular level enzyme activity of the trained muscles, which lead to improved fat oxidation and decreased accumulation of lactate during submaximal effort [8,10]. High-intensity endurance training leads to strengthening of the left ventricle wall and, thus, increases in stroke volume and cardiac output [9]. Together, these adaptations lead to improved endurance performance and are also associated with decreased FATM [33,34], as observed in the present study. 

On the other hand, progressive strength training leading to neuromuscular adaptations, e.g., an improved rate of force production, might develop endurance performance through improved exercise economy and sprinting ability [35]. Some concerns related to an interference effect of combined strength and endurance training have been presented, but they have mainly addressed the possible attenuating training effect on maximal strength development [19]. Only one study [36] has found a detrimental effect of combined training on aerobic fitness. More recent reviews have concluded that combined strength and endurance training improves aerobic capacity to the same extent and decreases fat mass even more than either training mode performed independently [19]. In the present study, the same absolute number (n = 10) of soldiers in the group of strength emphasized training and in the group of evenly balanced strength and endurance training improved their endurance performance during the study (Figure 2). Combined training might, therefore, be a superior training model for soldiers when compared to strength or endurance training only [6].

Previous studies have shown that endurance performance of soldiers is susceptible to decline during deployment [37,38,39], which might be due to detraining. It has been shown that already a few weeks of reduction in the training frequency or complete detraining can lead to a significant decrease in aerobic fitness, both in highly trained and recreationally active participants [40]. In the military context, Dyrstad et al. [37] found that the average aerobic fitness of deployed Norwegian soldiers decreased during a 12-month operation in Kosovo. However, soldiers who reported active participation in endurance training during the deployment, actually improved their aerobic capacity by 3.5% [37]. In the previous international military deployment study, Sharp et al. [39] found that soldiers in the two highest pre-deployment aerobic fitness quartiles decreased their endurance performance during a 9-month follow-up in Afghanistan, while no changes were observed in soldiers in the initially lowest fitness quartiles. Similar findings have been reported by Warr et al. [24] who found that endurance training performed at least three times a week was adequate to maintain or improve the aerobic fitness of soldiers during deployment. The previous findings support the present results, suggesting that increased endurance training frequency/volume would likely have reduced the number of soldiers with low training response. It is also important to note that individual training history should be taken into account when implementing training plans for soldiers.

Indeed, the reduced endurance performance in the LR group might have occurred simply because the total training volume was too low for the maintenance of their baseline aerobic fitness. A recent study [41] investigated adaptations to a 6-week endurance training program with a training frequency varying from one to five times per week. In the first part of the study, participants performing a lower number of training sessions were more likely to be determined as the “non-responders”. For example, 81% of the participants who trained once a week decreased their endurance performance, whereas the respective proportion in the group of four weekly training sessions was only 18%. In the second part of the intervention, the non-responders completed two additional weekly training sessions for another six weeks. After the second part of the study, it was found that training induced positive adaptations in all participants [41]. In the present study, soldiers who improved their 3000-m running time during the study period were able to maintain their pre-deployment endurance training frequency, whereas the endurance training frequency of the LoR group decreased during the operation. In addition, the decrease in the endurance training frequency from the pre-deployment level was associated with an increase in 3000-m time during deployment. Despite the good training facilities, the motivation of some soldiers for physical training might have been suppressed by the continuous maintenance of vigilance and 24-h shiftwork when compared to the situation before the deployment. Therefore, some obligatory physical training should be considered to maintain minimum a physical training volume of the unmotivated soldiers.

The present study has several strengths and limitations. First, there is a limited number of studies which have been conducted in the actual area of international military operation. In most of the previous studies, the measurements have been performed in homelands, before and after the deployment, and thus, the transport as well as the delay between measurements and the deployment might have influenced the results. In the present study, all measurements were conducted in the deployment area during the crisis-management operation. However, implementing the study in the middle of the crisis management operation limited the possibility to select the best measurement methods and caused challenges to the logistics of the measurement devices as well as study personnel. Due to the priority of operative duties, all soldiers were not able to participate in every measurement, and thus, the number of soldiers was reduced in some of the tests. The same explanation might, at least partly, explain the discrepancy between interview and diary-based training frequencies. Except for patrolling and other observational duties, the soldiers mainly lived inside the military base and were served the same food during the follow-up. Furthermore, the present body composition and blood biomarker results did not reflect disturbances in hormonal balance either in the HiR or LoR group. These findings are supported by previously published results of rather low physical activity and work load [26], as well as well-maintained energy balance [27] during the same crisis management operation. Thus, there were no environmental or physiological barriers for the training adaptations during the operation. 

## 5. Conclusions

High level of strength and endurance capacity forms the cornerstones of soldier’s physical performance. Based on the present findings, soldiers who are more likely in a risk to decrease aerobic fitness during prolonged military operations are leaner and fitter in terms of lower body strength and power. The emphasis of combined strength and endurance training of the deployed soldiers should be varied individually and task-specifically. The volume of endurance training should be maintained, at least, at the level preceding the operation to attenuate performance decrements. On the other hand, continuous strength training is also important in order to maintain the necessary levels of strength and power performances, and it also has likely some positive additive effects on endurance performance. Finally, increases in fat mass should be avoided for preventing decrements in endurance performance and operational readiness.

## Figures and Tables

**Table 1:** Group comparison of baseline characteristics, in terms of mean (SD).

	High-Responders	Low-Responders	*p*
n	25	24	
Age (years)	31.2 (7.9)	28.7 (9.4)	0.089
Stature (cm)	179.4 (5.4)	181.4 (6.9)	0.37
Body mass (kg)	79.3 (7.8)	79.7 (8.9)	0.79
Body mass index	24.6 (2.0)	24.2 (2.2)	0.28
Muscle mass (kg)	38.0 (3.9)	40.3 (4.1)	0.046
Fat mass (kg)	12.8 (3.6)	9.6 (5.7)	<0.001
Maximal isometric force of the lower body (N)	3959 (532)	4564 (1116)	0.049
Maximal isometric force of the upper body (N)	1139 (235)	1204 (223)	0.28
Sit-ups (repetitions in 1 min)	42.8 (10.5)	46.7 (8.5)	0.20
Push-ups (repetitions in 1 min)	37.4 (11.7)	43.5 (13.2)	0.053
Pull-ups (repetition maximum)	8.6 (4.9)	10.8 (5.3)	0.10
Standing long jump (cm)	226.7 (16.4)	241.5 (27.4)	0.016
Military simulation test (s)	155.8 (23.1)	143.2 (24.2)	0.028
Serum testosterone (nmol·L^−1^)	16.1 (4.3)	16.1 (5.7)	0.71
Serum sex-hormone binding globulin (nmol·L^−1^)	31.4 (9.9)	33.2 (14.1)	0.82
Serum insulin-like growth factor-1 (pmol·L^−1^)	26.2 (8.8)	29.5 (11.0)	0.21
Serum cortisol (nmol·L^−1^)	420.6 (108.7)	440.4 (78.7)	0.63
Interview-based endurance training (times/week) *	2.34 (1.40)	2.58 (1.58)	0.66
Interview-based strength training (times/week) *	1.79 (1.41)	2.90 (1.18)	0.008
* Interviewed before the operation.			

**Table 2:** Group comparison in physical training and relative changes in measured variables during the operation, mean (SD).

	High-Responders	Low-Responders	*p*
n	25	24	
Training variables during the operation			
Endurance training (times/week)	1.7 (0.80)	1.9 (2.8)	0.22
Strength training (times/week)	1.3 (0.7)	2.1 (2.4)	0.052
Total training (times/week)	3.0 (1.0)	4.0 (5.0)	1.00
Low-intensity endurance training (min/week)	61.7 (22.9)	52.0 (18.4)	0.17
Moderate-intensity endurance training (min/week)	51.3 (11.2)	45.9 (16.4)	0.31
High-intensity endurance training (min/week)	32.7 (18.7)	37.4 (11.6)	0.27
Lower body strength training (kg/week)	14,354 (6076)	19,489 (6202)	0.010
Upper body strength training (kg/week)	10,428 (3272)	12,226 (4084)	0.31
Interview based endurance training (times/week)	2.41 (1.01)	1.38 (1.06)	0.002
Interview based strength training (times/week)	1.94 (1.07)	2.73 (1.51)	0.067
Relative change (%)			
Body mass (%)	−1.0 (2.5)	2.3 (2.8)	<0.001
Body mass index (%)	−1.0 (2.5)	2.3 (2.8)	<0.001
Muscle mass (%)	0.5 (3.0)	1.4 (2.7)	0.16
Fat mass (%)	−7.6 (11.7)	14.2 (20.4)	<0.001
Maximal isometric force of the lower body (%)	16.5 (17.5)	7.8 (13.3)	0.26
Maximal isometric force of the upper body (%)	2.1 (5.7)	1.9 (9.2)	0.67
Sit-ups (%)	6.3 (16.0)	5.5 (11.9)	0.91
Push-ups (%)	27.7 (21.9)	11.7 (26.1)	0.004
Pull-ups (%)	40.0 (49.8)	42.6 (66.1)	0.79
Standing long jump (%)	0.6 (9.2)	−1.0 (4.0)	0.89
Military simulation test (%)	−13.6 (6.8)	−7.5 (6.5)	0.006
Serum testosterone (%)	10.3 (31.9)	18.2 (33.1)	0.35
Serum sex-hormone binding globulin (%)	−18.3 (35.1)	−21.5 (26.3)	0.35
Serum insulin-like growth factor-1 (%)	−2.4 (42.8)	−3.5 (37.2)	0.69
Serum cortisol (%)	0.53 (48.2)	−9.9 (34.4)	0.52
Interview based endurance training frequency (%)	27.9 (56.7)	−40.1 (64.2)	0.001
Interview based strength training frequency (%)	8.7 (61.7)	14.7 (101.0)	0.73

## Supplementary Materials

The following are available online at https://www.mdpi.com/1660-4601/17/5/1688/s1, Table S1: Group-wise weekly mean (±SD) and range of the training frequency and load during the operation.
