# Supplementary material for: Differences in Training Adaptations of Endurance Performance during Combined Strength and Endurance Training in a 6-Month Crisis Management Operation
Source: Int J Environ Res Public Health. 2020 Mar 5;17(5):1688. doi: 10.3390/ijerph17051688 (PMC7084630; doi:10.3390/ijerph17051688)
Supplement: Supplementary file 1 [file ijerph-17-01688-s001.pdf]

Supplemental material

Table S1. Group-wise weekly mean (±SD) and range of the training frequency and load during the operation

	SE			Se			Es		
	n	Mean (SD)	Range	n	Mean (SD)	Range	n	Mean (SD)	Range
Endurance training frequency	14	1.7 (0.6)	0.6-3.1	14	0.6 (0.4)	0.0-1.3	18	2.1 (0.8)	0.8-3.5
Strength training frequency	14	1.6 (0.7)	0.7-2.8	14	1.8 (0.4)	1.2-2.7	18	0.8 (0.4)	0.0-1.2
Total training frequency	14	3.3 (1.2)	1.2-5.0	14	2.4 (0.8)	1.4-4.0	18	2.8 (1.1)	0.8-4.4
Low-intensity endurance training volume (min)	14	58.4 (30.8)	30-151	11	51.4 (21.8)	30-93	16	72.9 (31.7)	36-144
Moderate-intensity endurance training volume (min)	12	51.2 (11.8)	36-75	6	40.2 (16.9)	24-72	17	46.9 (13.6)	27-67
High-intensity endurance training volume (min)	5	43.7 (23.3)	16-77	3	29.0 (8.2)	22-38	9	31.7 (10.8)	23-53
Lower body strength training load (kg)	14	14317 (6992)	3010-27491	13	16994 (7746)	4440-31060	16	15453 (6290)	4691-27720
Upper body strength training load (kg)	12	10798 (4496)	4240-20786	12	11305 (3330)	6197-17632	16	9968 (4018)	1800-17341
